# Low-dose ionizing radiation induces mitochondrial fusion and increases expression of mitochondrial complexes I and III in hippocampal neurons

**DOI:** 10.18632/oncotarget.5790

**Published:** 2015-09-22

**Authors:** Ling Chien, Wun-Ke Chen, Szu-Ting Liu, Chuang-Rung Chang, Mou-Chieh Kao, Kuan-Wei Chen, Shih-Che Chiu, Ming-Ling Hsu, I-Chou Hsiang, Yu-Jen Chen, Linyi Chen

**Affiliations:** ^1^ Institute of Molecular Medicine, National Tsing Hua University, Hsinchu, Taiwan, R.O.C.; ^2^ Department of Medical Science, National Tsing Hua University, Hsinchu, Taiwan, R.O.C.; ^3^ Center for Brain Research, National Tsing Hua University, Hsinchu, Taiwan, R.O.C.; ^4^ Department of Radiation Oncology, MacKay Memorial Hospital, Taipei, Taiwan, R.O.C.

**Keywords:** radiation therapy, hippocampal neurons, mitochondrial fusion, low dose radiation, Pathology Section

## Abstract

High energy ionizing radiation can cause DNA damage and cell death. During clinical radiation therapy, the radiation dose could range from 15 to 60 Gy depending on targets. While 2 Gy radiation has been shown to cause cancer cell death, studies also suggest a protective potential by low dose radiation. In this study, we examined the effect of 0.2-2 Gy radiation on hippocampal neurons. Low dose 0.2 Gy radiation treatment increased the levels of MTT. Since hippocampal neurons are post-mitotic, this result reveals a possibility that 0.2 Gy irradiation may increase mitochondrial activity to cope with stimuli. Maintaining neural plasticity is an energy-demanding process that requires high efficient mitochondrial function. We thus hypothesized that low dose radiation may regulate mitochondrial dynamics and function to ensure survival of neurons. Our results showed that five days after 0.2 Gy irradiation, no obvious changes on neuronal survival, neuronal synapses, membrane potential of mitochondria, reactive oxygen species levels, and mitochondrial DNA copy numbers. Interestingly, 0.2 Gy irradiation promoted the mitochondria fusion, resulting in part from the increased level of a mitochondrial fusion protein, Mfn2, and inhibition of Drp1 fission protein trafficking to the mitochondria. Accompanying with the increased mitochondrial fusion, the expressions of complexes I and III of the electron transport chain were also increased. These findings suggest that, hippocampal neurons undergo increased mitochondrial fusion to modulate cellular activity as an adaptive mechanism in response to low dose radiation.

## INTRODUCTION

Radiation therapy (RT) using ionizing radiation (IR) has been used on malignant glioma, aneurysm or acoustic neuroma [1, 2]. Several ways of RT, including stereotactic radiosurgery (SRS), conventional fractionated RT, and proton therapy, are considered depending on the diseases and conditions. However, employing RT on brain lesions also creates a dose distribution with gradual fall-off. While the target lesions receive therapeutic dose of RT, the surrounding brain tissue inevitably receives a gradient, generally low dose of IR by current standard dose-painting techniques. The dose that the off-target normal brain received could range from 0.1 to 20 Gy depending on the planned RT dose to targets. In most cases, the therapeutic dose is 2 Gy per fraction for conventional fractionated RT and 20 Gy in single fraction for SRS. The major toxicity concern is the cognitive deficits caused by RT. High energy IR has long been implicated in DNA damage and cell death. The total dose for radiation therapy ranges from 20 to 60 Gy depending on the pathological types of target lesions, treatment volumes and radiation techniques. Except that for stereotactic radiosurgery with single or hypofractionated radiotherapy, the conventional fractionated radiotherapy usually are composed of approximate 30 daily fractions with 1.8 to 2 Gy per fraction. Each course of conventional fractionated radiotherapy in general takes 6 to 7 weeks, rendering the accumulated effect of radiation underscored. It was estimated more than 200,000 patients receive partial large-field or whole-brain irradiation every year [3]. While radiation therapy for brain tumors aims to benefit patients, the side effect of the cognitive impairment, such as poor memory and injury of the hippocampus, has been reported [4-8]. Nonetheless, a number of reports implicate beneficial effects of low dose radiation [9, 10] and raise our interest to investigate whether low dose radiation has hormesis effect.

As the central dogma suggests that radiation would result in nuclear DNA damage, accumulating evidence supports an argument of nuclear damage being secondary to the damage of mitochondria [11-13]. It seems plausible that mitochondria DNAs (mtDNAs) are very susceptible to oxidative/radiation damage because they lack the protection by proteins and histones [11, 14]. As a result, mitochondria undergo compensatory fusion to remove the dysfunctional mtDNA and to maintain the respiratory function [15, 16]. Along the line, fusion of mitochondria has been demonstrated to protect against neurodegeneration [17]. The mitochondrial fusion proteins (Mitofusin proteins, MFNs) are large transmembrane GTPases, which bind and hydrolyze guanosine triphosphate (GTP) to produce energy [18, 19]. In mammals, the major proteins for fusion process have two isoforms, Mfn1 and Mfn2, located at the outer mitochondrial membrane [20]. In the brain, Mfn2 is the main mitochondrial fusion protein [17]. In contrast, mitochondrial fission is regulated by dynamin-related protein 1 (Drp1) and mitochondrial fission 1 (FIS1) in mammals. The structure of Drp1 contains an N-terminal GTPases and a C-terminal GTPase effector domain [21]. FIS1 is a mitochondrial outer membrane protein and is as a receptor for Drp1.

Given that biological effects of low dose RT distributed in brain remains unclear, we investigated the responses of neurons to RT from low dose to therapeutic dose levels. To our knowledge, the information of low dose RT on mitochondria dynamics of neurons is lacking. In this study, we tested the hypothesis that, in response to low dose radiation, hippocampal neurons may modulate mitochondrial dynamics as an adaptive mechanism to regulate neuronal survival.

## RESULTS

### Effect of radiation on the survival of hippocampal neurons

**Figure 1:**
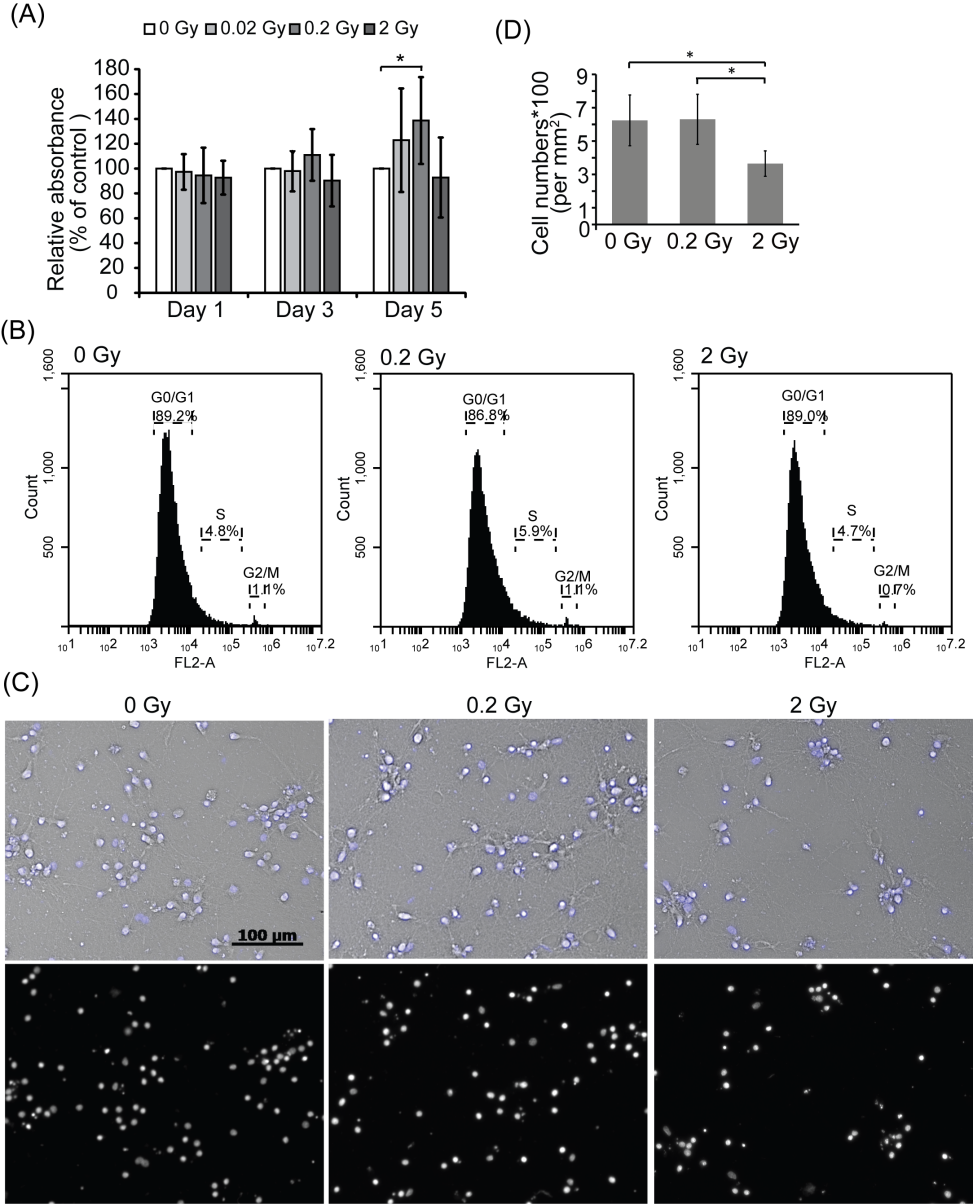
The level of MTT assays in 0.2 Gy-irradiated neurons was increased compared to control cells **A.** E18 hippocampal neurons were irradiated with 0, 0.02, 0.2, or 2 Gy radiation on DIV 7. Cell survival was determined via MTT assays 1, 3 or 5 days after radiation treatment. Values are mean ± S.E.M from three independent experiments. (*:*p* < 0.05, paired Student's *t* test) **B.** Neurons were treated as in **A.**. Five days after radiation, cells were harvested for cell cycle analysis. **C.** E18 hippocampal neurons were irradiated with 0, 0.2, or 2 Gy radiation on DIV 7. Five days after radiation treatment, cells were fixed and stained by DAPI. Images were taken using Zeiss Observer Z1 microscope with 10x (NA/0.3) objective. Scale bar = 100 μm. **D.** Cell number was measured. Values are mean ± S.E.M from three independent experiments. (*:*p* < 0.05, paired Student's *t* test).

To address the effect of radiation on hippocampal neurons, hippocampal neurons were isolated from embryonic day 18 (E18) rat and cultured *in vitro*. On day *in vitro* 7 (DIV 7), hippocampal neurons were irradiated with 0, 0.02, 0.2 or 2 Gy radiation. Cell viability was determined using MTT assays, 1, 3 or 5 days post-radiation. Five days after radiation, the OD565 in 0.2 Gy radiation-treated neurons was increased compared to control neurons (Fig. 1A). The results with 0.02-0.05 Gy radiation were rather variable, with averaged change of 10-18% (supplemental Table S1), which may reflect the limitation of the accelerator. Thus, 0.2 Gy is referred as low dose radiation in this study. MTT assays are often used as measurement for cell survival and/or cell proliferation. Neurons are post-mitotic and do not proliferate, thus the MTT data are not likely a result of neuronal proliferation. To confirm this assumption, cell cycle analysis was performed. As shown in Fig. 1B, radiation did not affect cell cycle progression of neurons. Although neurons are post-mitotic and are incapable of proliferation, it remains possible that 0.2 Gy radiation would increase neuron numbers through increasing differentiation of progenitor cells [22]. We thus examined whether low dose radiation may increase the numbers of hippocampal neurons. E18 hippocampal neurons were treated with 0, 0.2, or 2 Gy radiation on DIV 7. Five days after radiation, nuclei were stained with DAPI and counted (Fig. 1C and 1D). Comparing with control cells, cell number was decreased in 2 Gy radiation treated neurons. Cell number of 0.2 Gy-irradiated neurons was not affected. This result demonstrates that 0.2 Gy low dose radiation does not increase the number of E18 hippocampal neurons.

### 0.2 Gy radiation treatment has no effects on mitochondrial membrane potential, ROS level, mitochondrial DNA copy number but increases the level of the postsynaptic marker PSD95

**Figure 2:**
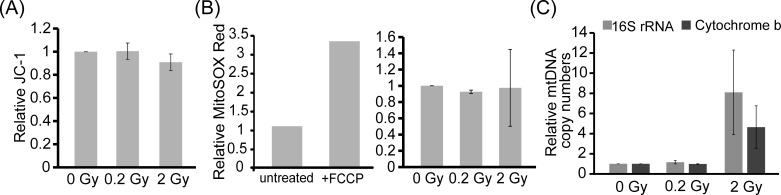
Radiation treatment did not have effects on mitochondrial membrane potential, ROS level, and mitochondrial DNA copy number **A.** E18 hippocampal neurons were irradiated with 0, 0.2, or 2 Gy radiation on DIV 7. Five days after radiation, neurons were collected and stained with JC-1 to measure mitochondrial membrane potential. **B.**-**C.** Neurons were treated as in **A.**. Five days after radiation, neurons were stained with MitoSOX Red and analyzed by BD Accuri C6 Flow Cytometer. Positive control on the left of **B.** showed increased MitoSOX Red for HUVEC cells treated with 40 μM carbonyl cyanide 4-(trifluoromethyoxy) phenylhydrazone (FCCP), an uncoupler of oxidative phosphorylation, for 30 min. Genomic DNA was extracted and mitochondrial DNA copy numbers were analyzed by Q-PCR. All quantified results are shown as mean ± S.E.M from three independent experiments.

While MTT assay is often used to detect the cell viability, the measured activity could also reflect mitochondrial activity [23]. We next determined whether low dose radiation may increase mitochondrial activity, mitochondrial membrane potential, mitochondrial reactive oxygen species (ROS) level and mtDNA copy number. Mitochondrial membrane potential (ΔΨm) is important for forming H^+^ electrochemical potential to generate ATP. JC-1 dye is a mitochondrial membrane potential indicator. In a healthy cell, JC-1 will aggregate and exhibit red fluorescence. When mitochondria are depolarized and ΔΨm values are decreased, JC-1 will exist as a monomer emitting green fluorescence. Neurons were treated with 0, 0.2, or 2 Gy radiation on DIV 7 and JC-1 dye was added to measure mitochondrial membrane potential via flow cytometry. The values of red/green fluoresce were normalized to control. As shown in Fig. 2A, comparing the mitochondrial membrane potential with or without radiation treatment, there is no significant difference among 0.2 or 2 Gy-irradiated neurons and the control neurons.

ROS are generated during mitochondrial respiration and may cause DNA damage. To determine whether radiation would affect ROS level, MitoSOX red was used to detect the ROS level. MitoSOX red is a mitochondrial superoxide indicator and can be oxidized by superoxide within mitochondria to produce red fluorescence. Neurons were treated with 0, 0.2, or 2 Gy radiation on DIV 7. Cells were collected five days after radiation treatment and stained with MitoSOX red to measure mitochondrial ROS level via flow cytometry. Comparing with 0 Gy, ROS level in 0.2 or 2 Gy-irradiated neurons was not significantly different from control neurons (Fig. 2B). A positive control was shown on the left.

mtDNA contains the genes required for respiratory activity and regulates mitochondrial ATP synthesis. An increase of mtDNA copy number is thought to be a compensatory effect for post-irradiated cells as part of an adaptive mechanism [24]. To determine whether radiation affects mtDNA copy number, neurons were treated with 0, 0.2, or 2 Gy radiation on DIV 7. Five days after radiation, the level of mitochondrial DNA copy number was quantified using semi-quantitative real-time PCR (Q-PCR) (Fig. 2C). 16S rRNA and cytochrome b on mtDNA serve as reference genes of mitochondria and β-actin serves as an internal control. The relative mtDNA copy number was normalized to β-actin. Mitochondrial copy number for 0.2 Gy-irradiated neurons was the same as non-irradiated neurons. On the other hand, mitochondrial copy number for 2 Gy-irradiated neurons was increased, though the increase was not statistically different. This result indicates that 2 Gy radiation may have generated cellular stress to neurons which is consistent with reduced neurons number upon 2 Gy radiation (Fig. 1C).

**Figure 3:**
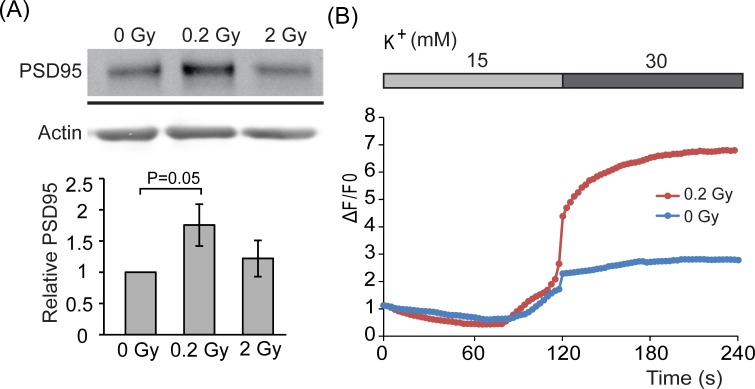
PSD95 expression levels in response to radiation **A.** E18 hippocampal neurons were irradiated with 0, 0.2, or 2 Gy radiation on DIV 7. After 5 days, cell lysates were collected and analyzed by western blotting using anti-PSD95 or actin antibody. Level of PSD95 was normalized to actin. Quantified results are shown as mean ± S.E.M from three independent experiments. **B.** E18 hippocampal neurons were irradiated with 0 or 0.2 Gy radiation on DIV 7. Five days after radiation, neurons were incubated with FM^®^ 4-64FX dye followed by increasing concentrations of K^+^ and images were taken. The intensity of fluorescence was quantified as described in material and methods. Blue line: 0 Gy radiation (*n* = 8) and red line: 0.2 Gy radiation (*n* = 22).

For the hippocampal neuron culture, the density of dendritic spines correlates with the number of synapses. To determine whether radiation affects dendritic spines, the level of PSD95, a postsynaptic marker, was determined. As shown in Fig. 3A, the level of PSD95 was higher in 0.2 Gy-irradiated neurons but not for 2 Gy-irradiation neurons. This result suggests that 0.2 Gy radiation may increase dendritic synapses to cope with the stimuli. Neurons communicate through the synaptic connections. The increase of dendritic synapses suggests increased connection and could represent increased activity. Thus, to evaluate the effect of low dose radiation on neuronal function, activity of synaptic vesicle cycling was monitored. To this end, hippocampal neurons were loaded with styryl dye FM4-64. Change of FM4-64 intensity at the soma area (mostly receiving information through dendrites) in response to depolarizing KCl solution was determined to represent synaptic activity. Neurons irradiated with 0.2 Gy showed increased uptake of dye compared to control neurons, suggestive of increased dendritic activity of neurons (Fig. 3B).

### 0.2 Gy radiation treatment promotes mitochondrial fusion and modulates mitochondrial complexes

**Figure 4:**
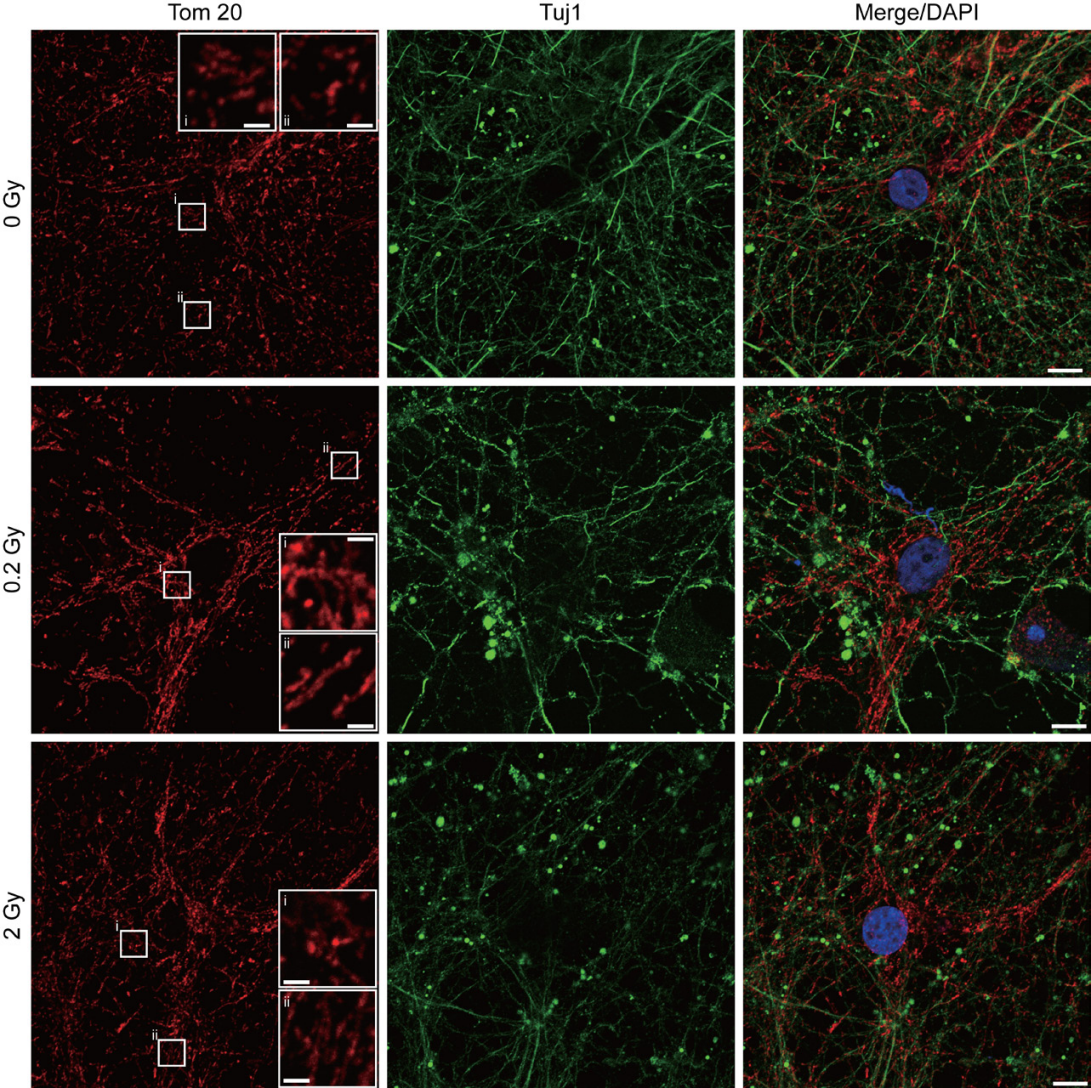
Radiation promotes mitochondrial fusion of hippocampal neurons E18 hippocampal neurons were irradiated with 0, 0.2, or 2 Gy radiation on DIV 7. After 5 days, cells were fixed and subjected to immunofluorescence staining with anti-Tom20 (Mitochondria, red) and Tuj1 (βIII-tubulin, green) antibodies. Nucleus was stained by DAPI (blue). The lower right panels of the Tom 20 staining represent magnifications of the areas indicated (i, ii). Scale bar = 10 μm. 20 μm for enlarged panels. Images were taken using Zeiss LSM780.

**Figure 5:**
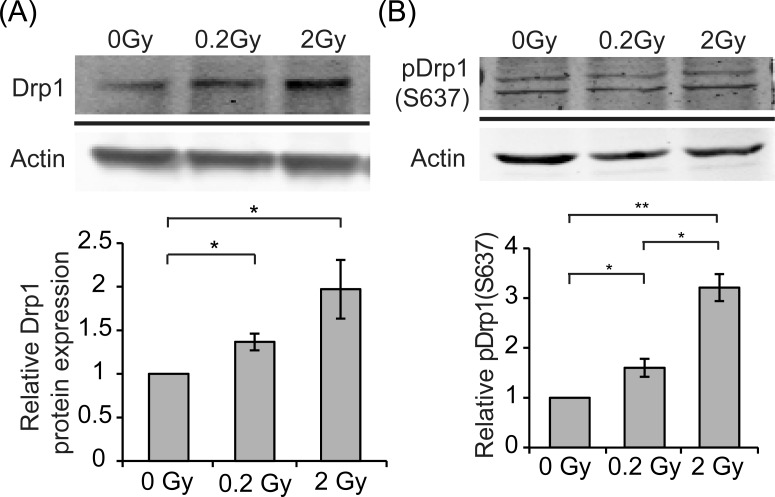
Radiation increases the level of Drp1 and pDrp1 E18 hippocampal neurons were irradiated with 0, 0.2, or 2 Gy radiation on DIV 7. Cell lysates were collected five days after radiation and analyzed by western blotting using anti-Drp1, pDrp1(S637), or actin antibody. Levels of Drp1 and pDrp1(S637) were normalized to actin for each experiment. Quantified results are shown as mean ± S.E.M from at least three independent experiments. (*:*p* < 0.05, **: *p*<0.01 paired Student's *t* test).

**Figure 6:**
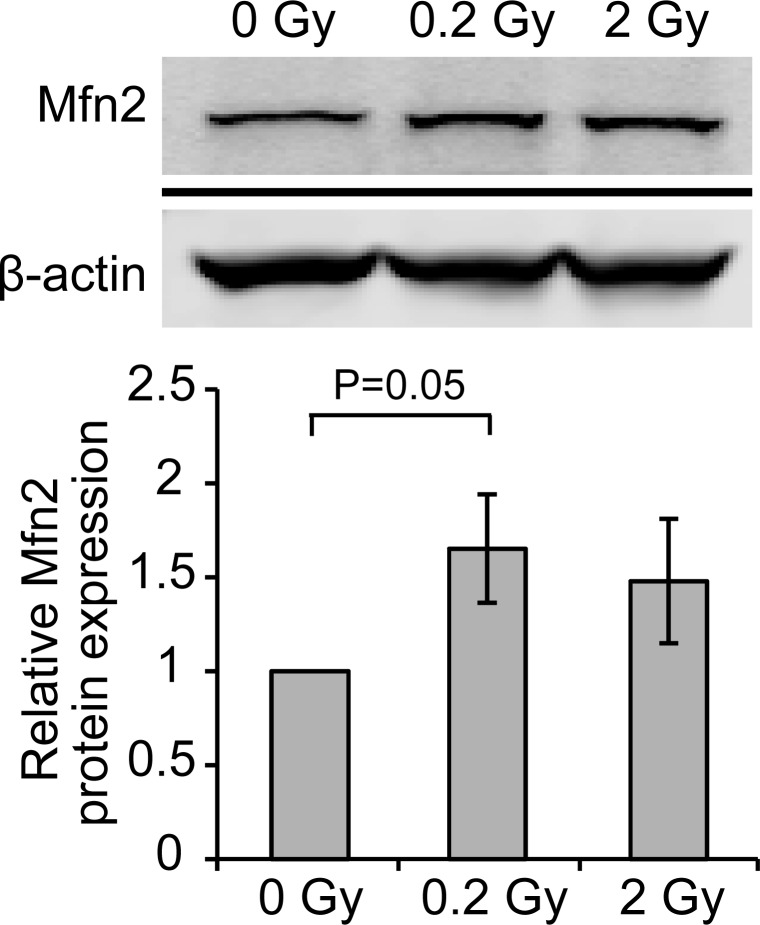
Radiation increases the level of Mfn2 E18 hippocampal neurons were irradiated with 0, 0.2, or 2 Gy radiation on DIV 7. Cell lysates were collected five days after radiation and analyzed by western blotting using anti-Mfn2 antibody. Actin was used as a loading control. Quantified results are shown as mean ± S.E.M from at least three independent experiments. (p value was calculated via, paired Student's *t* test).

Mitochondria supplies energy required during neuronal differentiation. To determine whether low dose radiation may change mitochondrial dynamics, neurons treated with 0, 0.2, or 2 Gy radiation were subjected to immunofluorescence staining of Tom20, a translocase of mitochondrial outer membrane, to mark mitochondria (Fig. 4). In response to 0.2 Gy radiation, morphology of mitochondria in neurons became longer/elongated compared to control neurons. 2 Gy irradiation also caused some degree of mitochondrial fusion but not to the degree observed in 0.2 Gy-irradiated neurons. This morphological change of mitochondria suggests a protective mechanism of neurons against radiation through promoting mitochondrial fusion. To determine the mechanism underlying radiation-induced mitochondrial fusion, the level Drp1, a mitochondrial fission protein, was examined via western blotting. As shown in Fig. 5A, the level of Drp1 was increased in response to 0.2 and 2 Gy irradiation. While the increase of Drp1 suggests an increased fission, interestingly, the relative level of pDrp1(S637) was also increased for 0.2 and 2 Gy-irradiated neurons compared to control neurons (Fig. 5B). Phosphorylation of Drp1 at Ser637 (S637) sequesters Drp1 in the cytoplasm and prevents its translocation to the mitochondria [25, 26]. Thus, the increased Drp1 for 0.2 Gy-irradiated neurons does not likely to lead to mitochondrial fission. In contrast, the expression of mitochondrial fusion protein, Mfn2, was increased specifically for 0.2 Gy-irradiated neurons (Fig. 6). This result is in line with the phenotype of increased mitochondrial fusion by 0.2 Gy-irradiation (Fig. 4). These findings suggest that 0.2 Gy low dose radiation promotes mitochondrial fusion to ensure neuronal survival.

**Figure 7:**
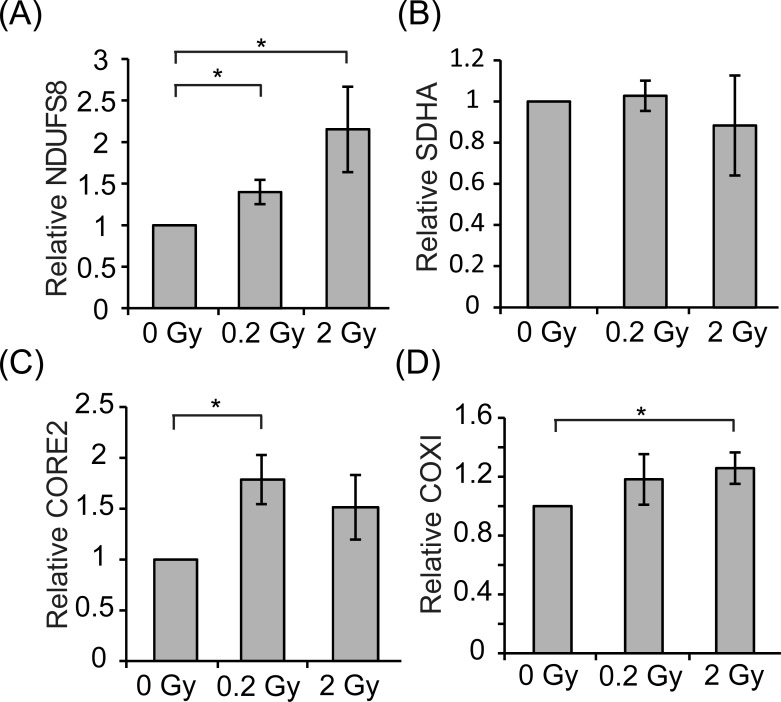
Protein expression of representative mitochondrial complexes E18 hippocampal neurons were irradiated with 0, 0.2, or 2 Gy radiation on DIV 7. After 5 days, cell lysates were collected and analyzed by western blotting using anti-NDUFS8, SDHA, CORE2, COX1 or actin antibody. Protein levels of NUFS8, SDHA, and COX1 were normalized to the level of actin for each experiment. Quantified results are shown as mean ± S.E.M from at least three independent experiments. (*:*p* < 0.05, paired Student's *t* test).

ATP synthesis requires five protein complexes localized on inner mitochondrial membrane for oxidative phosphorylation. The complex I accepts the electrons from the nicotinamide adenine dinucleotide and the complex II accepts the electrons from the flavin adenine dinucleotide. The electrons are passed to a lipid-soluble carrier coenzyme Q, as ubiquinone, and then are passed to complex III. Complex III transfers the electrons to a water-soluble electron carrier cytochrome c and the electrons are passed on to complex IV. The electrons combine H^+^ and O_2_ to form water at complex IV. Complex I, complex III and complex IV are coupled to H^+^ pumping from the matrix to intermembrane space to create the electrochemical gradient during the process of electron transfer. The electrochemical gradient is for Complex V to generate ATP. [27, 28]. To examined whether radiation affects the mitochondrial complexes, western blotting was performed to detect the expression of electron transport chain complexes, NDUFS8 (complex I), SDHA (complex II), CORE2 (complex III) and COX1 (complex IV). As shown in Fig. 7A and 7C, expressions of NDUFS8 and CORE2 were significantly induced by 0.2 Gy radiation. In contrast, expression of SDHA was not affected by radiation (Fig. 7B). Expression of NDUFS8 and COX1 were increased by 2 Gy irradiation (Fig. 7A and 7D). These results implicate that 0.2 Gy and 2 Gy radiation may increase mitochondrial activity and/or efficiency through promoting the fusion event.

## DISCUSSION

The biological effects of low-dose RT have been examined with controversial conclusions. Based on our previous work, 0.5 Gy radiation could enhance the immunotherapeutic effect of the autologous tumor lysate-pulsed dendritic cells against hepatoma cells. This immunogenic effect might be mediated through increased expression of MHC class ll and Fas at the cell surface of hepatoma cells [29]. In clinical practice, distribution of low dose RT to torso region has become a general phenomenon by dose-painting RT techniques. By using these advanced RT techniques, majority of the RT dose can be delivered to the intended tumor targets, leaving only low dose levels to surrounding normal tissues. We noted that the low dose level of 0.5 Gy delivered to abdomen could affect the systemic pharmacokinetic profile of chemotherapeutic drug 5-fluorouracil by declining the area under the concentration versus time curve (AUC) of 5-fluorouracil [30]. This abscopal response was demonstrated through increasing plasma level of matrix metalloproteinase-8 [31]. Intriguingly, we further discovered that low-dose RT of 0.5 Gy increased AUC of cisplatin in the plasma, supporting an abscopal effect of low dose RT [32]. Collectively, low-dose RT may have distinct biological effects that remain to be clarified when adopting advanced RT techniques in clinical practice.

Another issue regarding low dose radiation is the widely used computed tomography (CT) images in clinical diagnosis. Thus, re-evaluation of the effect of low dose radiation is of clinical importance. The embryonic and infant brains are especially sensitive to IR. However, not many studies address the effect of low dose radiation on embryonic or postnatal neurons. In this study, we demonstrate that low dose IR changes the dynamics of mitochondria of embryonic hippocampal neurons. Mitochondrial fission increases the number of mitochondria and removes damaged mitochondria through autophagy and apoptosis. Mitochondrial fusion, on the other hand, is required for mtDNA stability through sharing of components among mitochondria and to ensure mitochondrial homogeneity. Thus, a mechanism to protect cells from acute or low level of stress is through fusing functional and compromised mitochondria to exchange components among mitochondria [33]. Increased mitochondrial fusion can also increase mitochondrial mass to maximize oxidative capacity. Consistent with these findings, our results showed that low dose 0.2 Gy radiation increased mitochondrial fusion of hippocampal neurons, expressions of mitochondrial complexes I and III, and increased neuronal synapses. These findings suggest that low dose radiation may potentially protect neurons from stimulus through inducing mitochondrial fusion.

Neurodegenerative diseases, such as Charcot-Marie-Tooth disease and Alzheimer's disease (AD), are caused by fusion and fission defects of mitochondria [34-36]. In addition, mitochondrial fusion has been shown to protect against neurodegeneration [17, 37, 38]. Hippocampus is the region of the brain that suffers damage in AD patients. For AD patients, Aβ accumulation damages mitochondria and causes autophagy or apoptosis of hippocampal neurons. We also tested the possibility that low dose radiation may protect Aβ-treated neurons. Neurons were treated with 0, 5, 10 or 20 μM Aβ25-35 on DIV 7 to test the sensitivity of hippocampal neurons to Aβ25-35 (Fig. S1A). Based on the morphology and neuron numbers, 20 μM Aβ25-35 caused dramatic neuronal death. Thus, hippocampal neurons were then treated with the sub-lethal dosages, 0, 5 and 10 μM, of Aβ25-35 on DIV 7 and irradiated with 0, 0.2, or 2 Gy radiation on DIV 8. The morphology of hippocampal neurons treated with Aβ and radiation was similar five days after radiation (Fig. S1B). In addition, 1, 2, 5 days after radiation, apoptosis of cells was analyzed by Annexin V/PI staining and determined via flow cytometry (Fig. S2A). The percentages of apoptosis were not obviously affected after radiation treatment (Fig. S2B). Five days after 0.2 Gy radiation treatment, the percentages of cell survival were slightly increased compared to no radiation-treated control neurons (Fig. S2C). With one dose radiation, we did not find low dose radiation promoting survival of Aβ-treated hippocampal neurons. Nonetheless, it at least did not exacerbate the effect of Aβ. Whether cumulative effect of multiple sections of low dose radiation would be beneficial to Aβ-treated neurons remains to be determined.

0.2 Gy is used in CT scan and other medical diagnosis and is a dose potentially received by surrounding normal cells during brain tumor radiotherapy. The daily dose for treating patients suffering brain astrocytoma is 2 Gy. According to the dosimetry data by tomotherapy, the dose painting showed 0.2 Gy being the threshold dose of irradiation in the clinical practice (data not shown). These results could provide a promising strategy to preserve neuronal function upon radiation.

## MATERIALS AND METHODS

### Reagents

Hank's balanced salt solution (HBSS), Horse serum (HS), Minimum Essential medium (MEM), Neurobasal medium, Fetal bovine serum (FBS), L-glutamine (L-Gln), Penicillin-streptomycin, Antibiotic-antimycotic (AA), B27 supplement, Alex Fluor 488-conjuated IgG, Alex Fluor 555-conjuated IgG, DAPI, Prolong Gold, JC-1, Alexa Fluor® 488 Annexin V/Dead Cell Apoptosis Kit, TrypLE™ Express, MitoSOX™ Red and Power SYBR® Green PCR Master Mix were purchased from Life technologies (Carlsbad, CA). Cysteine, CaCl_2_, Papain, DNase I, Poly-L-lysine, Glutamate, Cytosine-1-β-D-arabinofuranoside (AraC), MTT powder and Bovine serum albumin (BSA) were purchased from Sigma (Saint Louis, MO). Anti-Tom 20 was purchased from Santa Cruz Biotechnology (Santa Cruz, CA). Anti-Drp1, anti-Mfn2 and anti-PSD95 were purchased from Cell signaling (Boston, MA). Anti-NDUFS8 was purchased from GeneTex (Irvine, CA). Wizard® Genomic DNA Purification Kit was purchased from Promega (Madison, WI). Triton X-100 was purchased from USB (Cleveland, Ohio). Anti-Tuj1 was purchased from Covance. Anti-SDHA, Anti-CORE2 and Anti-COX1 were purchased from Abcam. IRDye-conjugated secondary antibody was purchased from LI-COR Biosciences (Lincoln, NE). Rabbit polyclonal anti-pDrp1(S637) antibody was developed using phosphopeptide (Cys-Pro-Val-Ala-Arg-Lys-Leu-pSer637-Ala-Arg-Glu-Gln-Arg-Asp) of Drp1.

### Experimental animals and primary culture of hippocampal neurons

All experiments were conducted in accordance with the guidelines of the Laboratory Animal Center of National Tsing Hua University (NTHU). Animal use protocols were reviewed and approved by the NTHU Institutional Animal Care and Use Committee (Approval number 10214).

Primary hippocampal neurons were prepared from embryonic day 18 (E18) of Sprague-Dawley rats from BioLASCO Taiwan Co., Ltd. Hippocampal neurons were isolated as described previously [39, 40]. Cells were seeded on poly-L-lysine-coated plates (30 μg/ml poly-L-lysine in 0.15 M borated buffer, pH 8.4) and incubated at 37°C in 5% CO_2_. The first day that neurons cultured *in vitro* is defined as Day *in vitro* 0 (DIV0). Medium was changed to Neurobasal/Glutamate medium containing Neurobasal medium, 25 μM glutamate, 2% B27, 0.5 mM L-Gln and 50 units/ml penicillin-streptomycin on DIV1. Cells were treated with 10 μM AraC to inhibit glia cells growth on DIV2. On DIV3, medium was changed to Neurobasal/Glutamine medium containing Neurobasal medium, 2% B27, 0.5 mM L-Gln and 50 units/ml penicillin-streptomycin. After DIV3, Neurobasal/Glutamine medium was changed every 3 days.

### Radiation therapy via linear accelerator

Cells were irradiated with different doses of 6 MeV electron beam delivered by an Elekta Synergy Linear Accelerator (Elekta AB, Inc., SE. dose rate 200 MU/min) in a single fraction. Radiation on hippocampal neurons was performed at the MacKay memorial hospital, Hsinchu branch, Taiwan.

### MTT assays, cell number counting and cell cycle analysis

MTT (3-(4,5-dimethylthiazol-2-yl)-2,5-diphenyltetrazolium bromide) assays were performed according to the manufacture's suggestion. Briefly, MTT solution was added at final concentration of 0.5 mg/ml and incubated at 37°C for 4 hours. The OD565 value of formazan was measured using a plate reader. Results were expressed as percentage of untreated radiation neurons, assuming that the absorbance of untreated radiation neurons was 100%. Cells were stained by DAPI, mounted by Prolong Gold. Images were taken using Zeiss Observer Z1 microscope with 10x (NA/0.3) objective. Cell numbers were counted using ImageJ software. Cells with condensed nuclei were manually removed to exclude dying/dead cells.

For cell cycle analysis, hippocampal neurons were harvested by TrypLE, and fixed using 75% ethanol at 4°C overnight. Then, neurons were re-suspended in a solution containing 20 μg/ml propidium iodide and 100 μg/ml RNaseA in PBS at 37°C for 30 min. Neurons were then analyzed by BD Accuri C6 Flow Cytometry.

### Immunofluorescence, confocal microscopy and uptake of FM4-64 dye by hippocampal neurons

Cells were cultured on poly-L-lysine-coated coverslips. Cells were fixed by 4% paraformaldehyde for 15 minutes and permeabilized by 0.1% Triton X-100 for 10 minutes. Cell were incubated with 1% BSA/PBS, and then incubated with specific antibody. Anti-Tom 20 and anti-Tuj1 antibodies were used at dilutions of 1:200 and 1:500 in 1% BSA/PBS. Secondary antibodies used were Alex Fluor 488-conjuated IgG and Alex Fluor 555-conjuated IgG at dilutions of 1:1000 in 1% BSA/PBS. Nucleus was stained by DAPI at dilutions of 1:1000. Cells were mounted by Prolong Gold. Images were taken using Zeiss LSM780, confocal microscope system.

The dye uptake and analysis were modified based on Fairless et al [41]. The FM® 4-64FX dye (Invitrogen) for imaging was diluted in KCl/HBSS solution and added to hippocampal neurons at 10 μM final concentration. Neurons were irradiated by 0 or 0.2 Gy radiation on DIV 7. On DIV 12, FM® 4-64FX dye was loaded to neurons, images were taken using Zeiss Observer Z1 microscope time lapse imaging system with 20x objective. The basal level of K^+^ concentration was set as 5 mM and K^+^ concentration was increased to 15 mM and then to 30 mM. Fluorescence imaging was followed at exposure time of 300 ms; interval of 400 ms; and duration of 2 min. The intensity of FM® 4-64FX fluorescence was determined by Axiovision software (Zeiss) and the relative fluorescent changes (ΔF/F0) were calculated as the differences between fluorescence intensities (ΔF) divided by the average basal level intensity (F0).

### Immunoblotting

Cells were lysed in RIPA buffer (50 mM Tris-HCL, pH7.5, 1% Triton X-100, 150 mM NaCl, 2 mM EGTA) containing protease inhibitors [1 mM phenylmethylsulfonyl fluoride (PMSF), 1 mM Na_3_VO_4_, 10 ng/ml aprotinin and 10 ng/ml leupeptin (A+L)]. Lysates were then sonicated by using bioruptor sonication system (Bioruptor^®^ Plus, low spin, 30 sec/min for 5 min). Lysates of equal amount of proteins were separated by sodium dodecyl sulfate polyacrylamide gel electrophoresis (SDS-PAGE) and immunoblotted with the indicated primary antibodies followed by IRDye-conjugated secondary antibody incubation. The protein signal was detected using Odyssey infrared imaging system (LI-COR Bioscience).

### Measurement of mitochondrial membrane potential and mitochondrial reactive oxygen species level using flow cytometry

JC-1 dye is a mitochondrial membrane potential indicator. Cells were washed with PBS and stained with JC-1 (final concentration: 2 μM in Neurobasal medium) at 37°C for 30 minutes. Cells were harvested by TrypLE, centrifuged (500 xg, 5 minutes at 4°C) and re-suspended with PBS on ice. The samples were analyzed by BD Accuri C6 Flow Cytometry.

MitoSOX Red is a mitochondrial superoxide indicator. Cells were washed with PBS and stained with MitoSOX Red (5 μM in HBSS) at 37°C for 30 minutes. Cells were harvested by TrypLE, centrifuged (500 xg, 5 minutes at 4°C) and re-suspended with PBS on ice. The samples were analyzed by BD Accuri C6 Flow Cytometry.

### Genomic DNA extraction and measurement of mitochondrial DNA copy numbers

Cells were harvested, centrifuged (500 xg, 5 minutes at 4°C) and dissolved in EDTA/Nuclei Lysis Solution (0.5 M EDTA solution, pH 8.0 in Nuclei Lysis Solution) plus Proteinase K. After treating with RNaseA, samples were precipitated with isopropanol to isolate genomic DNA. β-actin region was amplified using primer pair 5′ AGC AGA TGT GGA TCA GCA AG 3′ and 5′ CAA TAA AGC CAT GCC AAA TG 3′. 16S rRNA region was amplified using primer pair 5′ TTG ATC AAC GGA CCA AGT TAC 3′ and 5′ CTG GAT TGC TCC GGT CTG A 3′. Cytochrome b region was amplified using primer pair 5′ CTT CTT CGC ATT CCA CTT CA 3′ and 5′ GGA TGG AAT GGG ATT TTG TC 3′.
